# Erector spinae plane block versus paravertebral block in analgesic outcomes following breast surgery

**DOI:** 10.1186/s12871-022-01950-9

**Published:** 2023-01-11

**Authors:** Ahmed M. Elewa, Mohammed Faisal, Folke Sjöberg, Mohamed E. Abuelnaga

**Affiliations:** 1 Department of Anaesthesia and intensive care, Faculty of Medicine, Suez Canal University, Ismailia, Egypt; 2 Department of Surgery, Faculty of Medicine, Suez Canal University hospital, Egypt, General Surgery department, Sahlgrenska University Hospital, Gothenburg, Sweden; 3 Department of Biomedical and Clinical Sciences (BKV), The Burn Center Linköping University Hospital, Linköping, Sweden; 4 Department of Anaesthesia and Intensive Care, Faculty of Medicine, Suez Canal University, Ard Elgameiat, P.O. Box:41522, Ismailia, Egypt

**Keywords:** Erector spinae plane block, Modified radical mastectomy, Paravertebral block

## Abstract

This article represents the response to the inquiries adopted by Dr. Raghuraman M Sethuraman, M.D., regarding our recently published study which compared the erector spinae plane block (ESPB) versus paravertebral block (PVB) regarding postoperative analgesic consumption following breast surgeries (Elewa et al, BMC Anesthesiol 22: 1-9, 2022). We would like to introduce our appreciation and gratitude to the author for his interest in our work, despite being inaccurate in some of his comments.

Dear Editor,

We read carefully all comments done by Dr. Raghuraman M Sethuraman, M.D. regarding our work [1]. We thank him for his comments which will help us to explain some important ideas.

First, the author stated that ESPB is only a technical modification of PVB, and it does not cover supraclavicular nerves, pectoral nerves, or other brachial plexus nerves, but is easier to perform and safer when compared to PVB, hence; does not require much expertise.

We believe that ESPB cannot be considered a modification for PVB. From our point of view, several differences exist between ESPB and PVB. During the ESPB the local anesthetic solution is injected in the interfascial plane between the erector spinae muscle and the transverse process, During PVB, the local anesthetic solution is injected into the paravertebral space between the superior costo-transverse ligament and the parietal pleura [2].

During PVB, there is an anteromedial spread of the local anesthetic into the paravertebral space combined with a lateral intercostal spread. The ventral rami of the spinal nerve and the sympathetic ganglion are usually involved in a successful PVB, and epidural spread through the intervertebral foramen is often noted [3].

During ESPB, there is a significant spread of the local anesthetic in the fascial layer and the back muscles [4, 5].

There is growing clinical evidence that ESPB can involve the ventral rami and sympathetic nerves, yielding analgesia for visceral pain and some sympathetically mediated symptoms, and even motor blockade [6–9].

Magnetic resonance imaging of living subjects has demonstrated contrast medium spread into the paravertebral and even epidural spaces across multi-segmental levels with the ESP block [10, 11].

The author claimed that ESPB does not cover the brachial plexus nerves, which is not accurate. Several clinical studies have investigated the analgesic effect of ESPB for upper extremity surgery and have yielded positive results [12–16].

Second, the author claimed that we incorrectly stated that “ESPB can be utilized in low-resourced facilities” as the resources required are the same for both, despite he stated in the same paragraph that ESPB is more easily performed and safer than PVB, so, it does not require much expertise.

In our institute, we used to perform ESPB guided with ultrasound or fluoroscopy as well as by using a blind technique in case of unavailability of guiding methods. We believe that ESPB is a simple technique to the degree that it can be performed guided by anatomical landmarks as described by previous publications [17–19], so it can be used in low-resource hospitals.

Third, the author stated that we incorrectly cited the article by Gürkan et al [20], by writing the year of publication in 2017 instead of 2020. Unfortunately, this is right, it was written by mistake because we have cited two different publications for the same author (reference numbers 11 and 21 in our publication) [1].

Lastly, reference number 26 in our publication was written by mistake in the text instead of reference number 25 because of an unintended typing error.

## Data Availability

Not Applicable.

## Abbreviations

ESPB: Erector spinae plane block
PVB: Paravertebral block
